# Improved diagnosis of faecal incontinence in the gastroenterology domain: The time to act has come

**DOI:** 10.1002/ueg2.12546

**Published:** 2024-02-08

**Authors:** Edoardo Vincenzo Savarino, Giuseppe Chiarioni

**Affiliations:** ^1^ Gastroenterology Unit Azienda Ospedale Università di Padova Padua Italy; ^2^ Department of Surgery, Oncology and Gastroenterology University of Padua Padua Italy; ^3^ Il Cerchio Med Healthcare Verona Centre Verona Italy; ^4^ UNC Center for Functional GI and Motility Disorders University of North Carolina at Chapel Hill Chapel Hill North Carolina USA

**Keywords:** biofeedback, constipation, diarrhoea, disorders of gut‐brain interaction, faecal incontinence, inflammatory bowel disease, irritable bowel syndrome

Faecal incontinence (FI) refers to recurring, uncontrolled leaking of solid or liquid stool for a period of at least 3 months in an individual with a developmental age of at least 4 years, according to the Rome Foundation for disorders of gut‐brain interaction (DGBI).^1^ The definition has been rehearsed by the recently conveyed United European Gastroenterology Committee aiming to address all issues involved in FI management from diagnosis to treatment of difficult cases by means of an inclusive, multispecialty approach.^2^ Of note, this cooperative project was promoted by both health care providers and patient's organisation sensitive to FI.^2^

FI is a prevalent symptom ranging from 7% to 15% in community‐dwelling individuals with a devastating impact on quality of life (QOL).^3^, ^4^, ^5^ Moreover, FI may affect up to 50% of institutionalised patients.^4^ Unexpectedly, FI goes often unrecognised because up to 70% of patients are either embarrassed to report it or poorly questioned by physicians for the challenges it poses.^1^, ^2^, ^3^, ^4^ Indeed, ‘faecal incontinence’ is a term used among physicians and researchers to improve communication.^4^ However, it is poorly understood by some patients and is avoided by others for the stigma perception.^4^, ^6^ Many patients prefer the term ‘accidental bowel leakage’ to describe FI.^6^ To this regard, the Guidelines on FI states: ‘Health care professionals should enquire about patient's symptoms in a sensitive manner and determine whether the patient suffers from FI’.^2^ Moreover, the Guidelines suggest that the patient may hide the symptom and consult for a different complain (e.g. diarrhoea).^2^ Likewise, the recent European Guidelines for functional bowel disorders with diarrhoea were also concordant on ‘questioning all patients with chronic diarrhoea about faecal incontinence with appropriate phrasing for it’.^7^ Regretfully, both recommendations were solely based on expert opinion.^2^, ^7^ However, the diagnosis of FI relies on patient's report and no confirming test is required.^2^ Since half of the patients consulting for FI would respond to simple diet and behaviour advise, Gastroenterologists should feel frustrated by missing the diagnosis for the ‘don't tell, don't ask policy’.^2^, ^7^

The value of patients' consulting for DGBI is strongly supported by the manuscript of Li and colleagues published in the present issue of the United European Gastroenterology Journal.^8^ DGBI, formerly known as functional gastrointestinal disorders, are chronic conditions characterised by persistent and/or recurring gastrointestinal symptoms that occur in the absence of organic disease.^9^ DGBI such as the irritable bowel syndrome, are highly prevalent worldwide and burdensome yet not thoroughly addressed in medical training.^9^ In their retrospective cohort trial Li et al aimed to report on the prevalence and nature of DGBI on 249 mostly female FI patients referred for anorectal physiology to a tertiary Neurogastroenterology Care. The symptom impact on general wellbeing and QOL was extensively evaluated by dedicated questionnaires including: Rome III Integrative Questionnaire, FI Severity Index, Hospital Anxiety and Depression Scale, and SF‐36. Three patients only were found to have symptoms limited to FI. In the remaining 246 individuals the mean number of additional, concurrent DGBI was 2.2 +/− 1.57 per patient (mean +/− S.D.). The most common concurrent diagnosis was functional defecation disorders (36.5%), followed by irritable bowel syndrome (27.1%), and then functional constipation (20.7). Moreover, a greater number of comorbid DGBI in FI was associated with worse FI symptoms, higher anxiety, and depression scores, and worse QOL, but did not appear correlated with disordered anorectal physiology. The study has some limitations for being retrospective, run on selected, mostly female patients, and for missing reports on stool consistency by the Bristol stool scale.^8^, ^10^ However, it enlarges and strengthens recent findings from a UK referral Centre on the clinical relevance of unexpected, coexistent FI and constipation.^11^ Moreover, it relies on a standardised anorectal manometry protocol providing solid evidence against the speculation of FI as simply due to a weak anal sphincter.^12^ A complex interplay is involved in the maintenance of continence including faecal consistency, sensory‐motor bowel and pelvic floor function, and their mostly relevant brain control as suggested by the efficacy of both biofeedback and sacral neuromodulation in refractory FI patients.^2^, ^13^, ^14^ Moreover, recent data from the inflammatory bowel disease (IBD) world showed the relevance of FI in driving the response to therapy and the QOL of the subjects, adding concerns for the missed FI diagnosis in the gastroenterology domain.^15^

In conclusion, research data are compelling and stand clear: the times to consider faecal incontinence as the silent affliction of the Gastroenterology domain are over. All patients consulting a Gastroenterologist for any DGBI or chronic diarrhoeal disorder such as IBD should be proactively screened for comorbid FI by sensitive phrasing. Once the diagnosis is disclosed, the European Guidelines for FI are available to provide skilled management^2^

## CONFLICT OF INTEREST STATEMENT

**Edoardo Vincenzo Savarino** has served as speaker for Abbvie, Agave, AGPharma, Alfasigma, Aurora Pharma, CaDiGroup, Celltrion, Dr Falk, EG Stada Group, Fenix Pharma, Fresenius Kabi, Galapagos, Janssen, JB Pharmaceuticals, Innovamedica/Adacyte, Malesci, Mayoly Biohealth, Omega Pharma, Pfizer, Reckitt Benckiser, Sandoz, SILA, Sofar, Takeda, Tillots, Unifarco; has served as consultant for Abbvie, Agave, Alfasigma, Biogen, Bristol‐Myers Squibb, Celltrion, Diadema Farmaceutici, Dr. Falk, Fenix Pharma, Fresenius Kabi, Janssen, JB Pharmaceuticals, Merck & Co, Nestlè, Reckitt Benckiser, Regeneron, Sanofi, SILA, Sofar, Synformulas GmbH, Takeda, Unifarco; he received research support from Pfizer, Reckitt Benckiser, SILA, Sofar, Unifarco, Zeta Farmaceutici. **Giuseppe Chiarioni** has received fees for advisory/speaker board by: Coloplast, EBNeuro, Errekappa Farmaceutici, Laborie, Mayoli.

## Data Availability

Data available on request from the submitting author.
